# Supplementary material for: A retrospective cohort study evaluating correlates of deep tissue infections among patients enrolled in opioid agonist treatment using administrative data in Ontario, Canada
Source: PLoS One. 2020 Apr 24;15(4):e0232191. doi: 10.1371/journal.pone.0232191 (PMC7182261; doi:10.1371/journal.pone.0232191)
Supplement: S1 Appendix — (DOCX) [file pone.0232191.s001.docx]

Appendix A: Summary of Study Variables, Type, Sources and Variable Format for Analysis

| **Variable** | **Type** | **Source** | **Analysis Format** |
| --- | --- | --- | --- |
| COVARIATES |  |  |  |
| Age | Categorical | RPDB | 15-24  25-34  35-44  45-54  55-64  >65* |
| Sex | Dichotomous | RPDB | Male*  Female |
| Income | Categorical | RPDB (ICES macro) | 1 (lowest income)  2  3  4  5* |
| Location of residence | Categorical | RPDB | Northern rural  Northern urban  Southern rural  Southern urban* |
| HIV status | Dichotomous | OHIP | Yes  No* |
| OUTCOMES |  |  |  |
| All-cause mortality | Dichotomous | RPDB | Yes  No |
| Frequent emergency department visits | Dichotomous | NACRS | Yes  No |
| Hospitalizations | Dichotomous | DAD | Yes  No |
| All-cause mortality | Dichotomous | RPDB | Yes  No |
