# Supplementary material for: A retrospective cohort study evaluating correlates of deep tissue infections among patients enrolled in opioid agonist treatment using administrative data in Ontario, Canada
Source: PLoS One. 2020 Apr 24;15(4):e0232191. doi: 10.1371/journal.pone.0232191 (PMC7182261; doi:10.1371/journal.pone.0232191)
Supplement: Attachment — Submitted filename: Response to reviewers.docx [file pone.0232191.s002.docx]

| **Reviewer question/comment** | **Author Response** | **Reference in Document** |
| --- | --- | --- |
| **Editorial requirements** | | |
| Could you therefore please include the title page into the beginning of your manuscript file itself, listing all authors and affiliations. | The title page has been added to the manuscript | Page 1 in Manuscript, line 1-30 |
| i) Please confirm that this does not alter your adherence to all PLOS ONE policies on sharing data and materials, by including the following statement: "This does not alter our adherence to  PLOS ONE policies on sharing data and materials.” (as detailed online in our guide for authors <http://journals.plos.org/plosone/s/competing-interests>).  If there are restrictions on sharing of data and/or materials, please state these. Please note that we cannot proceed with consideration of your article until this information has been declared.   ii) Please include your updated Competing Interests statement in your cover letter; we will change the online submission form on your behalf | The required statement has been added to the competing interest statement | Added to cover letter as requested |
| Please include your tables as part of your main manuscript and remove the individual files. Please note that supplementary tables (should remain/ be uploaded) as separate "supporting information" files  We note you have included tables to which you do not refer in the text of your manuscript. Please ensure that you refer to Tables 1 and 2 in your text; if accepted, production will need this reference to link the reader to the Tables. | Tables have been included as part of the main manuscript and references to the tables are added in the main text | In text reference:  Table 1, Table 2,Table 3, Figure 1 Figure 2 |
| **Reviewer 1 comments** | | |
| **Introduction** Page 2, last paragraph – “We also hypothesize that individual patient characteristics such as age, gender and location of residence, are correlated with DTIs.” - The authors do not justify or explain what research informs this hypothesis - what have studies said about sex/gender, age and residence and DTIs? In general, the authors need to make it more clear what the gaps in the research are concerning risk factors for DTIs and how this paper addresses this gap. Useful references not included in the paper are: (Larney, Peacock, Mathers, Hickman, & Degenhardt, 2017) and (Dahlman, Berge, Björkman, Nilsson, & Håkansson, 2018). |  | Thank you for the suggested references. Please see changes in the Manuscript document, page 3, line 67-70, in text reference: *“Moreover, in a recent systematic review, Larney et al.”* |
| **Results** There is no paragraph on Sample Characteristics. Also, did the authors have a breakdown of the number of injecting drug users and non-injecting drug users in the sample? | We did not have a break down of injection and non-injection drug users | The paragraph on sample characteristics is on page 12, line 265-271I added a sub title to clarify, in text reference: “*A total of 55,924 patients were enrolled in”* |
| A brief description of the DTIs that are examined would be useful for those readers not familiar with these infections. |  | Brief description provided on page 11, line 242-251 in text reference: *“We included three distinct DTIs…”* |
| **Results** line 195-201 – Also include the n (%) for ‘one or more DTIs’.  Put in the n (%) for females (line 199), location and income groups, HIV and all cause mortality (200-201).  Line 229 – spell it out how OxyContin relates to your findings  line 206 – make it clear that you are reporting the multivariable analysis – e.g. “In the multivariable analysis …” |  | Page 12, line 266 in text reference: *“…6,784 (12.1%) of patients had one or more DTIs…”*  Page 12, line 268-269, in text reference: *“…of patients with DTI (37.9) than no DTI (34.8).”*  Page 16, line 310-327, in text reference: “*Between 2006 and 2011, Oxycodone became much more available…”*  Page 14, line 284, in text reference*:* “In the multivariate analysis, it was observed…” |
| Methods Check through the use of abbreviations – sometimes an abbreviation was not given in full – e.g. page 3 line 60 – ICES; page 4 line 67 - OHIP. |  | Page 5, line 98-99, in-text reference*:* ***“****ICES, formally known as the Institute for Clinical and Evaluative Sciences,…”*  Page 6, in text reference: “…billing data from the Ontario Health Insurance Plan (OHIP) database” |
| Page 4 line 73 - Information on databases and study variables are available. – Where are they available. |  | Appendix A, page 6, line 120, in text reference**: *“****Information on databases and study variables are available in Appendix A.”* |
| Page 4 Cohort Definition Line 76-81: A brief definition of the codes used would be useful – maybe in a Table. |  | See table 1, line 139 |
| Figure 1 Use footnotes to explain the abbreviations. I’m unclear about the exclusion criteria as it reads – it looks like a mix of inclusion and exclusion criteria. |  | Footnotes inserted under Figure 1, page 5, line 106-111, and language adjusted in cohort definition page 6, line 127-137, in text reference: *“A set of exclusion criteria was applied…”* |
| Line 258 onwards – Limitations: The authors highlight the limitations of using health administrative data for data analysis and briefly mentioned gaps in the data – they could also mention at line 263 other important variables e.g. mode of drug taking (injecting versus non-injecting), homelessness, access or engagement with other harm reduction (e.g. sterile needles/syringes, wipes). There are limitations in only having patient characteristic data and not data on drug taking and harm reduction behaviour. |  | Page 19, line 159-162, in text reference: *“There are also limitations in only having patient characteristic data and not data on drug taking and harm reduction…”* |
| **Reviewer 2 comments** | | |
| I think it needs you need to define at the outset that the study population is only those enrolled in treatment at some point. Therefore, this is really a different study than what you would typically expect. I'm assuming this population was chosen as a convenience sample? if there is another reason why those enrolled in treatment were selected that needs to be articulated at the outset. |  | Population parameter is defined in the introduction, page 3, line 170-172, in text reference: *“This study will focus on determining the incidence of IE, OM, and SA among individuals in OAT in Ontario…”*  Rationale for only including OAT patients is provided on page 4, line 183-186, in text reference: ***“****The number of people in OAT is commonly used as a proxy measure for estimating the prevalence of OUD in a population since it is difficult to estimate the true prevalence of patients not seeking treatment”* |
| In general, I have not really seen use of the phrase "DTI". SSTI, which is used in IDSA guidelines and throughout the literature is what I see more commonly. For your consideration. | We use “DTI” since we also included osteomyelitis septic arthritis which affects the bones and joints |  |
| Though perhaps obvious to some readers, I would recommend naming at some point the fact that OAT enrollment has been proven to decrease frequency of IDU but that many people continue to have IDU while on therapy. | Thank you for this recommendation | Page 11 and 12, line 224-226, in text reference: *“Previous studies have demonstrated that OAT enrollment has been proven to decrease the frequency of…”* |
| In lines 27 through 28 you use mortality data to highlight the opioid use epidemic. I understands data on number of people actually using are hard to come by as compared to mortality data, but one does not exactly equal the other. Lansky PLOS ONE 2014 has a helpful accounting of US based numbers. Do comparable Canadian data exist? If so, would be helpful to cite this. | I have not found recent Canadian data on people using opioids. A study published in 2009 estimated the number of people using prescription opioids non-medically between 2003-2005 (Popova, 2009). However, this would be a stark underestimation of the current number. Alternatively, estimates in Canada have been based on people who access OAT |  |
| Lines 143 -Are you able to better characterize patient HIV characteristics. You talk about numbers with HIV --do you have data on who at least had a documented HIV test within a defined period (1 year, 3 years). Were there people with positive HIV tests,.Again, the methods here really appear to select for a patient population that is disparate from the most high risk for DTIs (not in OAT treatment, HIV+ or HIV status undefined, etc). |  | More details are provided on HIV status data on page 10 and 11, line 199-207, in text reference: ***“****HIV can be an indication of the degree of morbidity of patients with OUD…”* |
| Lines 150, here as well as elsewhere you use the phrase "opioid user", which is generally not the preferred nomenclature to reference this patient population. I would recommend PWID - "persons who inject drugs", which is the phrase used by CDC, WHO, etc. |  | Thank you for this recommendation, language was adjusted throughout the text |
| Lines 180 Recommend outlining the names of the different DTI names rather than just the codes. |  | Brief description provided on page 11, line 242-250, in text reference: *“We included three distinct DTIs…”* |
| Lines 247 -This sentence could use references, the phrase "giving clean needles" is an oversimplification of what is provided at syringe exchange programs, as provision of clean tourniquets, alcohol pads, sterile water etc all contribute to decreased DTI risk. Would also utilize the phrase "harm reduction" as this is really the umbrella concept you are referencing here |  | Page 19, line 338-341, in text reference:” *Such harm reduction strategies…”* |
