# Supplementary material for: A retrospective cohort study evaluating correlates of deep tissue infections among patients enrolled in opioid agonist treatment using administrative data in Ontario, Canada
Source: PLoS One. 2020 Apr 24;15(4):e0232191. doi: 10.1371/journal.pone.0232191 (PMC7182261; doi:10.1371/journal.pone.0232191)
Supplement: Attachment — Submitted filename: Response to reviewers.docx [file pone.0232191.s003.docx]

# Response to reviewers

Thank you for your comments. Please see table below in response to the reviewers’ comments

| **Reviewer question/comment** | **Author Response** | **Reference in Manuscript (Track Changes)** |
| --- | --- | --- |
| There are too many abbreviations and acronyms used throughout the paper (e.g. OHIP, DTI, RPDB, IE, OM, SA, etc.) which could frustrate the reader.  Consider spelling out these terms instead | This is a fair comment. I have updated the document |  |
| Line 75 - Authors need to provide more supporting information as to why they hypothesize that females are at higher risk of DTI.  I believe this is stated in the methods (lines 158-159). | I apologize for making this unclear, two additional references were added to support the hypothesis:  Meyers, et al. (2018) and  Hayashi et al. (2016) | Line 76 |
| Line 77:  A reference is needed for this sentence. | Thank you, two references have been added to support the statement. | Line 78 |
| Line 134: If no one was excluded because there n=0 patients, then this should be deleted. | This was deleted. | Line 133-134 |
| Line 153 (and elsewhere):  "Ratio measures of effect" is not standard terminology.  Please change to "relative measure of association" throughout | The wording was adjusted throughout. |  |
| Line 195:  Why were those with missing income quintile classified as the lowest income group? | I apologize for making this unclear. | Line 134-195 |
| Line 210:  Overdose should be one word. | Thank you, this was corrected | Line 209 |
| Lines 258 - 260.  For the logistic regression modeling, was there more than one observation per patient in the analysis?  If so, how was this accounted for?  Usually generalized estimating equations or generalized linear mixed modeling is conducted but this is not clear if these methods were used. | For the logistic regression modeling, there was only one observation per patient (i.e., a diagnostic code for a deep tissue infection appeared in their record or not), therefore we were not looking for an average response, we are trying to predict the effect multiple covariates on one given patient. I apologize for making this unclear. It is now clarified in the text | Line 241-254 |
| Figure 2:  The number of cases is not particularly meaningful.  Please report the "rate" (i.e. per 1,000 population) | Thank you for highlighting. A new Figure was uploaded to reflect comments. | Line 86 |
| Lines 284 - 287:  With the referent group being >65, it appears that the odds ratios increase as age increases.  Why is this? | The >65 group was chosen as the reference group since it had the lowest proportion of patients with deep tissue infections, and is the smallest age group. This may be due to premature mortality in this population.  The odds ration increases because patients have more time in their lives (more years of drug use, etc.) as they get older. | Line 335-337 |
| Lines 293 - 295:  Please double check all confidence intervals.  It appears that some are not correct (23.07 - 6.98). | Thank you for highlighting. This was a typo that was corrected | Line 294 |
| Table 3:  Please indicate the reference group with either "Ref" or "1.0".  Do not leave the row blank. |  | Table 3 Line 301 |
| Lines 332 - 336:  Sentence is too long. Please break into two to increase readability. |  | Line 336-338 |
